# T5 Tracking Cardiac Output During Burn Resuscitation via Pulse Wave Analysis

**DOI:** 10.1093/jbcr/irac012.004

**Published:** 2022-03-23

**Authors:** Yi-Ming Kao, Ghazal ArabiDarrehDor, Mary A Oliver, Adam D Reese, John W Keyloun, Kevin K Chung, Lauren T Moffatt, Jeffrey W Shupp, Jin-Oh Hahn, David M Burmeister

**Affiliations:** University of Maryland, College Park, College Park, Maryland; University of Maryland, College Park, College Park, Maryland; Burn Center at MedStar Washington Hospital Center, Washington DC, District of Columbia; Burn Center at MedStar Washington Hospital Center, Washington DC, District of Columbia; Burn Center at MedStar Washington Hospital Center, Washington DC, District of Columbia; Uniformed Services University, Bethesda, Maryland; Burn Center at MedStar Washington Hospital Center, Washington DC, District of Columbia; MedStar Washington Hospital Center, Washington DC, District of Columbia; University of Maryland, College Park, College Park, Maryland; Uniformed Services University, Bethesda, Maryland

## Abstract

**Introduction:**

Urine output (UOP) still remains the primary endpoint utilized as a surrogate for cardiac output (CO) and adequacy of perfusion during burn resuscitation. The role of arterial blood pressure (BP) waveform as a guiding tool for burn resuscitation has not been rigorously explored. As we move toward developing and validating novel endpoint to complement UOP, we compared the potential of pulse wave analysis (PWA) of arterial BP waveforms for estimating cardiac output (CO) and stroke volume (SV) in a large animal model of 40% total body surface area (TBSA) burns with varying resuscitation paradigms.

**Methods:**

Anesthetized swine were instrumented and hemorrhaged 15% of their blood volume, and sustained a 40% TBSA full-thickness contact burn with aluminum billets. Animals were kept in a surgical ICU setting overnight, during which anesthesia was maintained with a combination of propofol, ketamine, and fentanyl. Animals were randomized to 3 different intravenous fluid (lactated Ringer’s, LR) levels: under resuscitation with no IV fluids, adequate resuscitation protocolized with a clinical decision support tool, or over resuscitation with a starting rate of 500mL/hour. We computed 20 surrogate measures of CO and SV (10 each) via PWA of arterial BP, and calibrated them to reference CO via thermo-dilution and SV on a subject-by-subject basis. Surrogate performance was quantified in terms of correlation coefficient and root-mean-squared error (RMSE) relative to reference CO and SV.

**Results:**

Animals received 0±0, 4.4±1.6, and 9.1±1.3 mL/kg/% TBSA in the under, adequate, and over resuscitation groups (p=0.0036), respectively. Among the ten surrogate measures of CO and SV, 4 surrogates had positive proportionality to reference CO and SV consistently. The best surrogate measure of CO and SV was [mean BP-diastolic BP]/[heart period] and [mean BP-diastolic BP], respectively, which yielded r value of 0.82+/-0.19 and RMSE of 0.23+/-0.14 lpm for CO and r value of 0.85+/-0.17 and RMSE of 2.7+/-2.0 ml for SV (Fig. 1).

**Conclusions:**

The initial results suggest that PWA-based surrogates of CO and SV have the potential to track reference CO and SV in extremes of resuscitation post-burn. The current model produces under-resuscitated, adequately resuscitated, and over-resuscitated animals, making it possible to rigorously examine the efficacy of PWA-based tracking of CO and SV in a wide spectrum of burn resuscitation scenarios. Hence, PWA may provide metrics useful for developing surrogates of CO and SV as novel treatment endpoints of burn resuscitation that indicate perfusion and volume status of burn injury patients.

